# Effect of different cuff widths on the motor nerve conduction of the median nerve: an experimental study

**DOI:** 10.1186/1749-799X-3-1

**Published:** 2008-01-09

**Authors:** Parul Mittal, Shweta Shenoy, Jaspal S Sandhu

**Affiliations:** 1 Department of Sports Medicine and Physiotherapy, Guru Nanak Dev University, Amritsar, India

## Abstract

**Background:**

A bloodless operative field is considered mandatory for most surgical procedures on the upper and lower extremity. This is accomplished by using either an Esmarch bandage or a pneumatic tourniquet, but a number of complications are associated with both. Nerve palsy is one of the most frequently encountered complications of this procedure. Wider cuffs have been found to cause reduced risk of tourniquet induced injury to the underlying soft tissues than the narrower ones due to the fact that lower occlusion pressures are caused by the former. To address and investigate this question, conduction in the median nerve has been measured proximal to tourniquet as well as distal to the tourniquet. Parameters of nerve conduction measured are nerve conduction velocity, latency and amplitude.

**Methods:**

Sphygmomanometer cuffs with widths 14 cm and 7 cm were applied to the upper extremities of 20 healthy, normotensive volunteers (9 males and 11 females with age ranging from 22 to 27). Systolic blood pressure was measured first and then the cuff was inflated to about 20–30 mm Hg above it and was kept inflated for 15 minutes. Recordings were done prior to, for the period of tourniquet inflation, and following release of the tourniquet.

**Results:**

Nerve conduction was found to be more severely affected by the 14 cm cuff than the 7 cm cuff.

**Conclusion:**

Wider cuffs resulted in more severe changes in the nerve. This brings us to the conclusion that though lower inflation pressures are required for the occlusion of the blood supply using wider cuffs, the nerve conduction is more severely affected by the wider ones. Both electrophysiological changes and occlusion pressure should be kept in mind while choosing the width of the cuff.

## Background

Many studies related to tourniquet have been conducted till date. Some of these experiments have investigated the various complications associated with tourniquet [1-5]. No doubt tourniquets are advantageous in providing a clear operative field view but it is also true that they provide this advantage at the risk of many complications. Rorabeck [6] stated that, out of many complications, the most frequent one that should be wholly prevented are the nerve palsies arising from the use of tourniquets.

After a surgery, it is easy to identify nerve palsy but it is difficult to attribute it to a single defined cause. In tourniquet induced nerve palsies, a few studies have attributed it to ischemia [7] and others have attributed it to deformation following pressure [8]. The width of the tourniquet is also an important factor in deciding the extent of these injuries. Wider tourniquet cuffs can achieve an effective arrest of the regional arterial circulation at sub systolic pressures of inflation [9], so it could be assumed that these must cause less intense injury to the underlying soft tissue structures in comparison with the narrow ones. This is just an assumption and has not been proved till now.

This study is an attempt to shed some light on the electrophysiological changes in the motor nerve of the median nerve when the tourniquet of different widths is used.

## Methods

The experimental protocol was reviewed and approved by the university research ethical committee. It was an experimental study with the same subject design {when one group of subjects is tested or measured on all the conditions and their performance compared it is known as Same Subject design, Related Subject design or Within Subject design} [10]. Subjects were thoroughly informed about the experiment and written informed consent was taken from them. Two sphygmomanometer cuffs of 7 cm and 14 cm width were used as tourniquet in the study. 20 normal subjects volunteered in the investigation, the purpose and procedure of which was explained to them in advance. There were 9 males and 11 females of age ranging from 22 to 27 [Mean age ± Standard Deviation [S.D.] = 24.45 ± 1.10].

After taking the blood pressure in a standard manner, the subject was placed in a supine position with the arm abducted to 90° and supported comfortably. Stimulating and recording electrodes were placed on the right upper extremity of each subject to stimulate the median nerve. Before placement, the skin below the electrodes was slightly abraded to reduce impedance. A ground electrode was fastened to a convenient site between the stimulating and recording electrode. The recording site was abductor policis brevis muscle. R1 and R2 electrodes were placed in such a way that R1 is placed over the muscle belly of abductor brevis muscle and R2 over the first metacarpophalngeal joint. Stimulation sites were axilla [proximal to tourniquet] and ante-cubital fossa [distal to tourniquet].

The resting motor nerve conduction velocity [MNCV], amplitude and latency measurements of the median nerve for each subject were carried out before the experiments. The decision to carry out the experiment at a particular time with either of the cuff was random and a gap of at least 24 hours was kept between the two experiments. The two cuffs were inflated to 20–30 mmHg above their respective systolic blood pressure ranging from 110 to 126 mmHg for 14 cm cuff and 140 to 166 mmHg for 7 cm cuff and were kept inflated for 15 minutes. An inflation pressure of 20–30 mmHg above the systolic blood pressure was used as the subjects included in the study were not anesthetized and the subjects would not have tolerated higher pressures than this. During this time period three recordings of the motor nerve conduction was taken by stimulating at both the axilla and the ante-cubital fossa. The first recording was done at 5^th ^minute of inflation, the second one at 10^th ^minute and the third at 15^th ^minute. The cuff was then deflated and again recording of same parameters were taken at 1^st ^minute, 2^nd ^minute, 3^rd ^minute, 4^th ^minute, 15^th ^minute and 30^th ^minute of post deflation, stimulating the same points.

The motor nerve conduction velocity before and following the application of the two cuffs was calculated by dividing the distance between the two stimulation sites by the difference in the onset latency proximal and distal to the cuff i.e.

CV (m/s) = distance (mm)/LAT_proxtocuff _- LAT_distaltocuff_.

The percentage of MNCV, amplitude and latency was calculated using the formula i.e. [value of each parameter at different time durations/baseline value]*100

Throughout the experiment, the room temperature was maintained between 23°C and 26°C with the help of air conditioning. Paired t-test was used to compare the changes in the nerve conduction parameters with the application of 2 cuffs. All values which appear with the mean values are standard deviations [S.D.].

## Results

The present study has demonstrated that the wider 14 cm cuff impairs conduction in the nerve more severely than the 7 cm cuff. Initially 2-way ANOVA with post hoc Tukey's Multiple Comparison test was applied to the obtained data. No statistically significant difference was obtained between the parameters compared for the two cuffs, so a more sensitive Paired t-test was applied and the results of the same are presented below.

### Conduction Velocity

**Figure 1:**
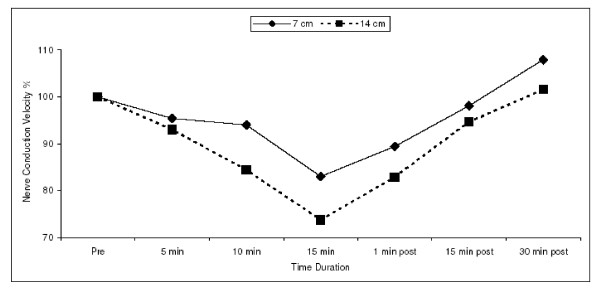
Mean values of the percentage MNCV at different time durations before and following the application of the 14 cm and 7 cm cuff. A decrease in MNCV is evident following application of both cuffs but as can be clearly seen that this decrease is more with the 14 cm cuff after 15 minutes of inflation of the cuff.

The decrease in MNCV was maximum with the 14 cm cuff. Though decrease in the conduction velocity occurred with both the 14 cm & the 7 cm cuff, the reduction was more with the former. After 5 minutes of inflation of the 14 cm cuff, MNCV was 93.01% ± 11.34 of its baseline value whereas with the 7 cm it was 95.40% ± 11.94. Though a decrease was evident, statistically this was not significant when the two tourniquets were compared. [Fig. 1]

The difference became statistically significant only after 10 minutes of inflation of the cuffs. At 10 minutes of inflation, when the percentage decrease in MNCV was compared for the two cuffs, a statistically significant difference was found with the p-value ≤ 0.03. With the 14 cm cuff the MNCV was 84.37% ± 10.15 of its baseline value after 10 minutes of inflation whereas with the 7 cm cuff the MNCV was 93.97% ± 16.10 of its baseline value. After 15 minutes of inflation also a significant difference was found in between the two cuffs with the significance level of p ≤ 0.04. At this time the MNCV was 73.73% ± 12.06 of its baseline value for 14 cm cuff whereas it was 82.96% ± 16.33 for the 7 cm cuff.

The release of the tourniquet allowed these values to return to normal, the amount of time required for the same varied i.e. the conduction velocity took about 15 minutes to return to normal with the 7 cm cuff whereas with the 14 cm cuff it was 30 minutes.

### Latency

**Figure 2:**
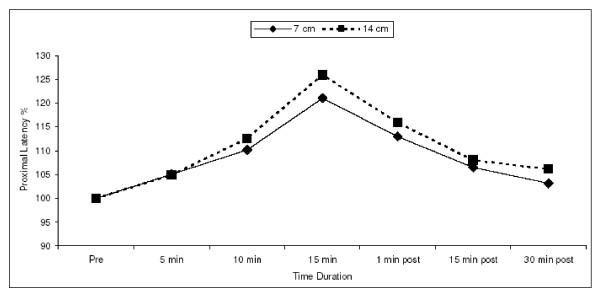
Plot of increase in the percentage latency measured proximal to the two cuffs with the wider cuff (14 cm) showing greater increase in the same during the 15 minutes of inflation of 14 cm cuff in comparison to the narrower one (7 cm).

As far as the onset latency is concerned, a prolongation in latency measured both distal & proximal to the tourniquet was found but the significant difference in this prolongation between the 2 cuffs was noted in the latency measured proximal to the cuff [Fig. 2]. A significant difference was obtained in the percentage latency measured proximally to the cuff at 15 minutes of inflation with p ≤ 0.02, whereas in the latency measured distal to the cuff no significant difference was found between the 2 cuffs at any of the time duration.

As soon as the cuff was removed, the value of latency started returning to normal with no significant difference between the two cuffs at 15 minutes of removal of the cuff.

### Amplitude

**Figure 3:**
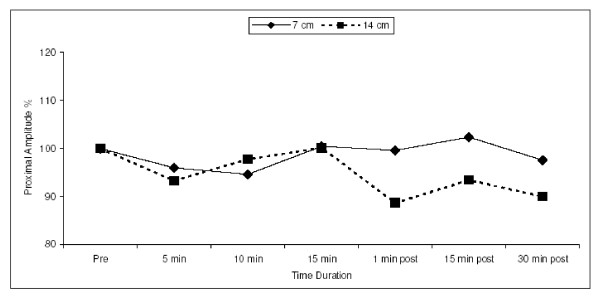
Figure showing the percentage amplitude change measured proximal to the two cuffs during the 15 minutes of inflation of the cuffs. The graph shows no definite pattern being followed by either of the two cuffs.

No significant difference was found in the percentage of amplitude, recorded either proximally or distally to the two cuffs, at different time durations with the 2 tourniquets. Also no defined pattern of increase or decrease in amplitude was found with the 2 cuffs [Fig. 3].

## Discussion

The primary aim of the study was to investigate the effect of different width cuffs on the motor nerve conduction of the median nerve. Graham et al.[9] in their study found that wide cuffs can reduce the risk of tourniquet induced injury to the underlying soft tissues by lowering the inflation pressure required to secure a bloodless field. The results of the present study contradict these findings by the fact that even though the amount of pressure required for occluding the blood supply was less with the 14 cm cuff in comparison to the 7 cm cuff in the present study, the changes in the motor nerve conduction was greater with the former.

The possible causative factor that resulted in the present results could be the area of the nerve being compressed by the tourniquet. As the area of the nerve compressed under the 14 cm cuff is more, this could have resulted in more severe changes in nerve conduction by the same. Denny Brown & Brenner [11] while comparing the effect of localized direct pressure and the effect of application of the sphygmomanometer cuff to surface of the limb found that even when the external pressure exerted by the sphygmomanometer cuff was sufficiently high to cause an effective internal pressure, failure was more rapid than when a corresponding local compression with the mercury bag was used.

Different studies have identified one of the two causal factors i.e. ischemia and mechanical pressure as the main reason leading to an injury to the structures underlying the tourniquet following a surgery. A few studies have mentioned ischemia to be the cause of the impaired conduction in the nerve [7] whereas others have mentioned deformation resulting from the pressure as the causal factor [8]. If the clinical aspect is considered then it is clear that both time duration and the inflation pressure of tourniquet associated with the surgical procedures will play an important and decisive role.

Mechanical pressures could be an unlikely explanation as enormous pressures were necessary to abolish conduction in excised frog nerve enclosed in an oxygenated pressure chamber [12]. Usually such high pressures are not encountered in surgical procedures, so anoxia of the larger area of the nerve could be hypothesized as the possible causal factor for the more severe impairment of nerve conduction with the wider 14 cm cuff. Also the inconsistent results in the amplitude could be because of the short duration for which the tourniquet was kept inflated in the present study, as a complete cessation in the conduction has been found only after about 30 minutes of inflation of the cuff [13,14].

The results of the present study suggest that the inflation pressure for occlusion of blood supply should not be the only factor while considering the safety of the width of the cuff as electrophysiological changes are equally important in deciding the appropriate width of cuff. Thus while choosing the appropriate width of the cuff both occlusion pressure and electrophysiological changes in nerve should be kept in mind, so that least damage could occur to the underlying structures.

In the present study, subjects recruited were not anesthetized and also the amount of inflation pressure and the time duration for which the cuffs were applied was also small in comparison to what occurs in routine surgical procedures so there was no question of damage occurring to the underlying structures as is evident by the complete recovery that occurred after the cuff was removed. Thus to generalize the findings of the present study, further studies can be carried with more number of different cuff widths and also with the tourniquet inflation time and occlusion pressure simulating the time duration and occlusion pressure of surgical procedures.

## Conclusion

Wider cuffs result in more severe changes in nerve conduction velocity than the narrow ones. This suggests that while choosing the appropriate width of the cuff, both occlusion pressure and electrophysiological changes in nerve should be kept in mind.

## Authors' contributions

PM carried out the data collection and drafted the manuscript. SS and JSS were the co-investigators in the study.
